# HIV-1 Drug Resistance Emergence among Breastfeeding Infants Born to HIV-Infected Mothers during a Single-Arm Trial of Triple-Antiretroviral Prophylaxis for Prevention of Mother-To-Child Transmission: A Secondary Analysis

**DOI:** 10.1371/journal.pmed.1000430

**Published:** 2011-03-29

**Authors:** Clement Zeh, Paul J. Weidle, Lillian Nafisa, Humphrey M. Lwamba, Jully Okonji, Emily Anyango, Philip Bondo, Rose Masaba, Mary Glenn Fowler, John N. Nkengasong, Michael C. Thigpen, Timothy Thomas

**Affiliations:** 1 Division of HIV/AIDS Prevention, US Centers for Disease Control and Prevention (CDC), Kisumu, Kenya; 2 Division of HIV/AIDS Prevention, National Center for HIV, Viral Hepatitis, STD, and TB Prevention, CDC, Atlanta, Georgia, United States of America; 3 Centre for Global Health Research, Kenya Medical Research Institute, Kisumu, Kenya; 4 Johns Hopkins Medical Institute, Department of Pathology, Baltimore, Maryland Onsite at Makerere University, Johns Hopkins University Research Collaboration, Kampala, Uganda; 5 Division of Global HIV/AIDS, Center for Global Health, CDC, Atlanta, Georgia, United States of America; National Institute of Child Health and Human Development, United States of America

## Abstract

**Background:**

Nevirapine and lamivudine given to mothers are transmitted to infants via breastfeeding in quantities sufficient to have biologic effects on the virus; this may lead to an increased risk of a breastfed infant's development of resistance to maternal antiretrovirals. The Kisumu Breastfeeding Study (KiBS), a single-arm open-label prevention of mother-to-child HIV transmission (PMTCT) trial, assessed the safety and efficacy of zidovudine, lamivudine, and either nevirapine or nelfinavir given to HIV-infected women from 34 wk gestation through 6 mo of breastfeeding. Here, we present findings from a KiBS trial secondary analysis that evaluated the emergence of maternal ARV-associated resistance among 32 HIV-infected breastfed infants.

**Methods and Findings:**

All infants in the cohort were tested for HIV infection using DNA PCR at multiple study visits during the 24 mo of the study, and plasma RNA viral load for all HIV-PCR–positive infants was evaluated retrospectively. Specimens from mothers and infants with viral load >1,000 copies/ml were tested for HIV drug resistance mutations. Overall, 32 infants were HIV infected by 24 mo of age, and of this group, 24 (75%) infants were HIV infected by 6 mo of age. Of the 24 infants infected by 6 mo, nine were born to mothers on a nelfinavir-based regimen, whereas the remaining 15 were born to mothers on a nevirapine-based regimen. All infants were also given single-dose nevirapine within 48 hours of birth. We detected genotypic resistance mutations in none of eight infants who were HIV-PCR positive by 2 wk of age (specimens from six infants were not amplifiable), for 30% (6/20) at 6 wk, 63% (14/22) positive at 14 wk, and 67% (16/24) at 6 mo post partum. Among the 16 infants with resistance mutations by 6 mo post partum, the common mutations were M184V and K103N, conferring resistance to lamivudine and nevirapine, respectively. Genotypic resistance was detected among 9/9 (100%) and 7/15 (47%) infected infants whose mothers were on nelfinavir and nevirapine, respectively. No mutations were detected among the eight infants infected after the breastfeeding period (age 6 mo).

**Conclusions:**

Emergence of HIV drug resistance mutations in HIV-infected infants occurred between 2 wk and 6 mo post partum, most likely because of exposure to maternal ARV drugs through breast milk. Our findings may impact the choice of regimen for ARV treatment of HIV-infected breastfeeding mothers and their infected infants.

**Trial Registration:**

ClinicalTrials.gov NCT00146380

*Please see later in the article for the Editors' Summary*

## Abstract 2

Analysis of a substudy of the Kisumu breastfeeding trial by Clement Zeh and colleagues reveals the emergence of HIV drug resistance in HIV-positive infants born to HIV-infected mothers treated with antiretroviral drugs.

## Editors' Summary

**Background:**

Globally, more than 2 million children are infected with the human immunodeficiency virus (HIV) that causes acquired immunodeficiency syndrome (AIDS), and half a million children are newly infected every year. These infections are mainly the result of mother-to-child transmission (MTCT) of HIV during pregnancy, labor and delivery, or through breastfeeding. MTCT can be greatly reduced by treating HIV-positive mothers and their babies with antiretroviral drugs (ARVs). Without ARVs, up to half of babies born to HIV-positive mothers become infected with HIV. This rate of transmission falls to below 5% if a combination of three ARVs is given to the mother throughout pregnancy. Unfortunately, this triple-ARV therapy is too expensive for use in the resource-limited countries where most MTCT occurs. Instead, many such countries have introduced simpler, shorter ARV regimens such as a daily dose of zidovudine (a nucleoside reverse transcriptase inhibitor or NRTI) given to HIV-positive women during late pregnancy coupled with single-dose nevirapine (a non-nucleoside reverse transcriptase inhibitor or NNRTI) at the onset of labor, zidovudine and lamivudine (another NRTI) during labor and delivery, and single-dose nevirapine given to the baby at birth.

**Why Was This Study Done?:**

More than 95% of HIV-exposed children are born in resource-limited settings where breastfeeding is the norm and is crucial for child survival even though it poses a risk of HIV transmission. Consequently, several recent studies have investigated whether MTCT can be further reduced by giving the mother ARVs while she is breastfeeding. In the Kisumu Breastfeeding Study (KiBS), for example, researchers assessed the effects of giving zidovudine, lamivudine, and either nevirapine or nelfinavir (a protease inhibitor) to HIV-infected women from 34 weeks of pregnancy through 6 months of breastfeeding. The results of KiBS indicate that this approach might be a safe, feasible way to reduce MTCT (see the accompanying paper by Thomas and colleagues). However, low amounts of nevirapine and lamivudine are transferred from mother to infant in breast milk and this exposure to ARVs could induce the development of resistance to ARVs among HIV-infected infants. In this KiBS substudy, the researchers investigate whether HIV drug resistance emerged in any of the HIV-positive infants in the parent study.

**What Did the Researchers Do and Find?:**

In KiBS, 32 infants were HIV-positive at 24 months old; 24 were HIV-positive at 6 months old when their mothers stopped taking ARVs and when breastfeeding was supposed to stop. The researchers analyzed blood samples taken from these infants at various ages and from their mothers for the presence of HIV drug resistance mutations (DNA changes that make HIV resistant to killing by ARVs). They detected no resistance mutations in samples taken from 2-week old HIV-positive infants or from the infants who became infected after the age of 6 months. However, they found resistance mutations in a third and two-thirds of samples taken from 6-week and 6-month old HIV-positive infants, respectively. The commonest mutations conferred resistance to lamivudine and nevirapine. Moreover, resistance mutations were present in samples taken from all the HIV-positive infants whose mothers who had received nelfinavir but in only half those taken from infants whose mothers who had received nevirapine. Finally, most of the mothers of HIV-positive infants had no HIV drug resistance mutations, and only one mother-infant pair had an overlapping pattern of HIV drug resistance mutations.

**What Do These Findings Mean?:**

These findings indicate that, in this KiBS substudy, the emergence of HIV drug resistance mutations in HIV-infected infants whose mothers were receiving ARVs occurred between 2 weeks and 6 months after birth. The pattern of mutations suggests that drug resistance most likely arose through exposure of the infants to low levels of ARVs in breast milk rather than through MTCT of drug-resistant virus. These findings need confirming but suggest that infants exposed to ARVs through breast milk—a situation that may become increasingly common given the reduction in MTCT seen in KiBS and other similar trials—should be carefully monitored for HIV infection. Providers should consider the mothers' regimen when choosing treatment for infants who are found to be HIV-infected despite maternal triple drug prophylaxis. Infants exposed to a maternal regimen with NNRTI drugs should receive first-line therapy with lopinavir/ritonavir, a protease inhibitor. The significance of the NRTI mutations such as M184V with regard to response to therapy needs further evaluation. The M184V mutation may result in hypersensitization to other NRTI drugs and delay or reverse zidovudine resistance. Given the limited availability of alternative drugs for infants in resource-limited settings, provision of the standard WHO-recommended first-line NRTI backbone, which includes 3TC, with enhanced monitoring of the infant to ensure virologic suppression, could be considered. Such an approach should reduce both illness and morbidity among infants who become HIV positive through breastfeeding.

**Additional Information:**

Please access these Web sites via the online version of this summary at http://dx.doi.org/ 10.1371/journal.pmed.1000430.

- The accompanying *PLoS Medicine* Research article by Thomas and colleagues describes the primary findings of the Kisumu Breastfeeding Study
- Information is available from the US National Institute of Allergy and Infectious Diseases on HIV infection and AIDS
- HIV InSite has comprehensive information on HIV/AIDS
- Information is available from Avert, an international AIDS charity, on many aspects of HIV/AIDS, including information on children, HIV, and AIDS and on preventing mother-to-child transmission of HIV (in English and Spanish)
- UNICEF also has information about children and HIV and AIDS (in several languages)
- The World Health organization has information on mother-to-child transmission of HIV (in several languages), and guidance on the use of ARVs for preventing MTCT

## Introduction

Worldwide in 2009, an estimated 370,000 infants acquired HIV infection primarily because of mother-to-child transmission (MTCT) in utero, during the peripartum period, or via breastfeeding [1],[2]. MTCT HIV transmission rates range from 25%–40% without antiretroviral medications, 8%–10% when short-course antiretrovirals are given during the peripartum period, and less than 5% when triple-combination maternal antiretroviral therapy is given throughout pregnancy [3]. In sub-Saharan Africa, progress has been made in the last few years toward expanding prevention of mother-to-child transmission (PMTCT) programs with simpler and shorter antiretroviral prophylactic regimens than those used in industrialized countries [4],[5]. Additionally, many country guidelines include treatment of eligible HIV-infected pregnant women with triple-combination antiretroviral therapy to improve the mother's health and for PMTCT. More than 95% of HIV-exposed children are born in resource-limited settings where breastfeeding is the norm [1]. Breastfeeding improves overall infant survival, and safe, feasible, and sustainable alternatives to breastfeeding are usually unavailable. According to WHO, breastfeeding in the first 6 mo of life provides 4- to 6-fold protection against mortality as opposed to no breastfeeding [6]–[8]. Early cessation of breastfeeding in an effort to limit the exposure to HIV-infected breast milk may be associated with increased infant morbidity and mortality [9]. The addition of antiretroviral therapy to HIV-infected mothers through the period of breastfeeding is one strategy to reduce HIV transmission via breast milk [10]. The potential mechanisms of protection would include both reduction in maternal viral load in breast milk as well as indirect infant prophylaxis by ingestion of antiretrovirals in breast milk, since several previous studies have shown that antiretroviral drugs (including zidovudine [ZDV], lamivudine [3TC], nevirapine [NVP], and efavirenz) administered to nursing women are present in their breast milk [11]–[13].

However, the presence of antiretroviral drugs in breast milk may have negative implications for infants who become infected before or during breastfeeding, because of the risk of acquisition of drug resistance caused by viral replication in the presence of low drug concentrations in the infant. To investigate this risk, we evaluated, in a secondary analysis, the emergence of drug resistance among infants born to HIV-infected mothers participating in the Kisumu Breastfeeding Study (KiBS)—a phase IIb PMTCT single-arm non-randomized trial that aimed to assess the safety and transmission rates of a triple-antiretroviral regimen consisting of ZDV, 3TC, and either NVP or nelfinavir (NFV) from 34 wk gestation through 6 mo post partum for PMTCT among HIV-infected breastfeeding mothers [14]. Evaluation of drug levels in the breast milk of a subset of mothers participating in the trial revealed the presence of 3TC and NVP in quantities sufficient to have therapeutic effect on HIV (such as prevention of HIV infection or partial suppression of HIV replication in infected infants and thus potential emergence of HIV drug resistance) [13]. In this secondary analysis, we evaluated the emergence of antiretroviral drug resistance mutations among HIV-infected infants, and ascertained whether resistance mutations developed as a result of exposure to low doses of maternal antiretroviral drugs via breastfeeding or whether they were acquired directly from the mother.

## Methods

Detailed description of the study design and methods is published [14]. In brief, enrollment was conducted between July 2003 and November 2006; follow-up was completed in February 2009. Women were recruited through the PMTCT programs in the antenatal clinics of the New Nyanza Provincial General Hospital and the Kisumu District Hospital. Pregnant women were invited to enroll if they were HIV positive and if, after receiving risk-benefit counseling on infant-feeding options, they indicated intent to breastfeed (Text S1).

The study intervention consisted of triple-combination maternal antiretroviral therapy (ZDV/3TC and either NVP or NFV) given from 34 wk gestation to 6 mo post partum. Women were instructed to exclusively breastfeed the infants for 5.5 mo and then wean over a 2-wk period with complete cessation of breastfeeding by 6 mo. Women who met World Health Organization (WHO) criteria for combination antiretroviral treatment (either with CD4 count <200/mm^3^, or WHO stage 3 or 4 HIV disease) [15] at initiation or subsequently through the study period were continued or reinitiated on antiretroviral therapy during the 24 mo of follow-up. Participants who did not meet treatment criteria had antiretroviral therapy discontinued at 6 mo post partum. Infants were initiated on antiretroviral treatment when they met WHO treatment criteria [15].

Between July 2003 and January 2005 participants received ZDV 300 mg and 3TC 150 mg in a fixed-dose combination tablet (Combivir, GlaxoSmithKline) taken twice a day and a NVP 200-mg tablet (Viramune, Boehringer Ingelheim) given in an incremental dose from one tablet daily for 2 wk and then one tablet twice a day for the duration of the study [16],[17]. Infants received a single 2-mg/kg dose of NVP liquid (Viramune, Boehringer Ingelheim) within 72 h of birth. In January 2005, the protocol was revised because of concerns of an increased risk of symptomatic liver toxicity for women on NVP with CD4>250 cells/µl [18]. Subsequently, participants with CD4≤250 cells/µl received ZDV, 3TC, and NVP as per the previous regimen, and participants with CD4>250 cells/µl received ZDV, 3TC as per the previous regimen and the protease inhibitor (PI), nelfinavir mesylate (NFV) 1,250 mg (as 5×250-mg tablets, Viracept, Hoffman La-Roche Ltd) twice daily.

### Ethics Statement

This study was approved by the Ethical Review Committee of the Kenyan Medical Research Institute as well as the Institutional Review Board of the US Centers for Disease Control and Prevention. Written informed consent was obtained from each participant prior to the study initiation.

### Specimen Collection and Laboratory Testing

Maternal and infant whole blood samples were collected in EDTA-treated anticoagulant vacutainer tubes (Becton Dickinson). Laboratory visits for blood collections were at delivery (between 0 and 7 d of birth), 2, 6, 14 wk, and 6, 9, 12, 18, and 24 mo. Infant HIV-PCR testing was done on dried blood spots using Roche DNA PCR version 1.5 (Roche Diagnostic System). Real-time HIV-PCR evaluation was done at 14 wk, and 6 and 9 mo. For those infants who were HIV-PCR positive, we retrospectively tested the earlier samples to determine the time of first positive HIV-PCR. HIV-1 plasma RNA viral load was quantified from all HIV-infected infant and mothers' specimens at multiple study visits, using the Roche Amplicor HIV-1 monitor version 1.5 (Roche Diagnostic System).

### Testing for HIV Drug Resistance and Viral Subtyping

HIV drug resistance mutation analyses of maternal and infant samples were performed using the FDA licensed ViroSeq HIV-1 genotyping system (Applied Biosystems) retrospectively, starting with the week 14 specimens from infants with viral load ≥1,000 copies/ml. For infants with detectable HIV drug resistance mutations at 14 wk, we sequenced the earlier samples (6, 2, and 0 wk) to determine time of first HIV drug resistance mutation. For infants with no HIV drug resistance mutations at 14 wk, we tested specimens at 6 mo. For infants who were first HIV-PCR positive after 14 wk, drug resistance mutation analysis was done on the first HIV-PCR–positive sample.

After genotyping on the ABI 3100 genetic analyzer, sample file analysis was performed by the Sequence analysis software version 3.7 (Applied Biosystems). The 1.5-kb *pol* region sequences were analyzed with ViroSeq HIV-1 genotyping analysis software version 2.6 to determine HIV drug resistance mutations, as per manufacturer's recommendations. HIV drug resistance mutations were confirmed to be consistent with the Stanford University HIV Drug Resistance Database and categorized according to the International AIDS Society-USA Drug Resistance Mutations Group December 2008 update [19]. To determine the extent of genetic diversity and the influence of specific viral subtype on resistance, the sequences were aligned using the Se-Al multiple sequence alignment program, Phylogenetic Analysis Using Parsimony (PAUP)* 4.0b using HIV reference sequences from GeneBank. HIV-1 inter-subtype recombination was analyzed using SimPlot software 2.0 and further analyzed by constructing a neighbor joining, bootstrapped phylogenetic tree, as implemented in PAUP [20].

## Results

### Study Population and HIV-PCR Results

**Figure 1:**
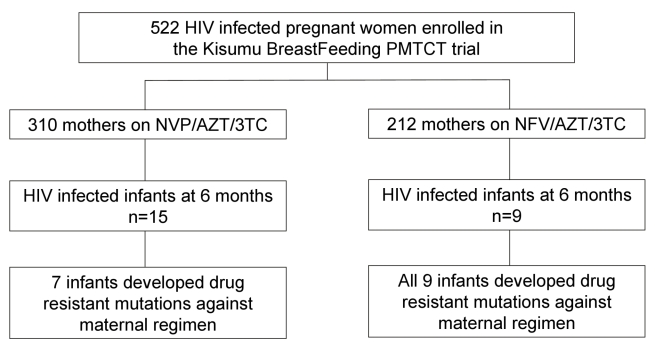
Cohort profile showing mothers enrollment, HIV transmission, and subsequent emergence of drug resistance in infants born to mothers in the NFV- and NVP-based regimens.

Between July 2003 and November 2006, we enrolled 522 pregnant women who met the eligibility criteria, of whom 21 withdrew and one woman died before delivery. The 500 women delivered 502 live born infants, including nine twin births, one triplet birth, and nine still births. By 24 mo, 32 infants (all singleton births) were HIV positive by PCR test with 24 of the infections having occurred within the first 6 mo of age. Of these 24 infants, 15 were exposed to a maternal NVP-based regimen, whereas nine were exposed to a maternal NFV-based regimen throughout the breastfeeding period (Figure 1). One mother changed regimen at week 14 post delivery from NVP to NFV because of liver toxicity. The rate and risk factors for HIV-MTCT transmissions in the KiBS trial are reported separately [14].

### Viral Load of the 12 Infants Who Were HIV-PCR Positive at Birth

**Figure 2:**
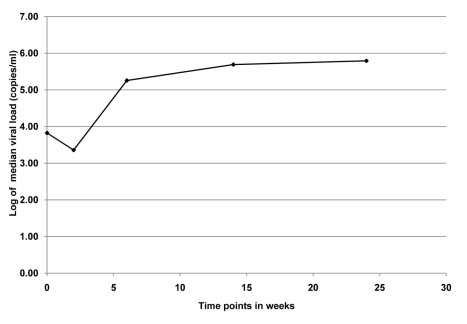
Median viral load for infants (*n* = 12) who were PCR positive at birth in the KiBS, Kisumu, Kenya, 2003–2008. The trend of median viral load at delivery was 2.8 log copies per ml, decreased ∼3-fold at 2 wk, increased ∼80-fold from 2 wk to 6 wk, and remained between 5 and 6 log copies per ml through 6 mo.

Among the 12 infants who were first HIV-PCR positive at delivery, we analyzed plasma viral load over the 6-mo breastfeeding period. Median viral load at delivery was 2.8 log copies per ml, decreased ∼3-fold at 2 wk, increased ∼80-fold from 2 wk to 6 wk, and remained between 5 and 6 log copies per ml through 6 mo (Figure 2).

### Development of HIV Drug Resistance

Among the 24 infants who were first HIV-PCR positive by 6 mo (the breastfeeding period), resistance mutations were detected in none of the eight with detectable viral load at delivery or 2 wk (six were not amplifiable), in six (30%) of 20 at 6 wk, in 14 (64%) of 22 at 14 wk, and in 16 (67%) of 24 at 6 mo (Table 1). Of the eight infants who were first HIV-PCR positive after 6 mo (after the intervention period), none developed drug resistance mutations. While only acknowledged by two mothers, continued breastfeeding is the most likely cause of infection after 6 mo. However, none of the eight mothers were taking antiretroviral therapy when the child was first diagnosed HIV-PCR positive.

**Table 1:** HIV-1 drug resistance emergence among breastfeeding infants in the Kisumu Breastfeeding (KiBS) trial, Kenya, 2003–2008.

Visit	First HIV-PCR Positive	Samples with Resistance (*n*)/Samples Tested (*n*)				
		0–2 wk	6 wk	14 wk	6 mo	>6 mo
Delivery	12	0/7	5/12	10/12	11/12	—
2 wk	2	0/1	0/2	1/2	1/2	—
6 wk	6	—	1/6	1/6	1/6	—
14 wk	2	—	—	2/2	2/2	—
6 mo	2	—	—	—	1/2	—
9–18 mo	8	—	—	—	—	0/8
**Total**	**32**	**0/8**	**6/20**	**14/22**	**16/24**	**0/8**

Age refers to scheduled visit age at which samples were obtained.

### HIV Drug Resistance Mutation Patterns by 6 mo

Of the 24 infants with HIV infection diagnosed by 6 mo, seven of 15 (47%) whose mothers were started on NVP and all nine (100%) of whose mothers were started on NFV, had drug resistance mutations (*p* = 0.0095, Fisher exact), but the 95% confidence interval for the difference was wide (28%–78%). Among the seven infected infants exposed to maternal NVP who developed resistance mutations, two (71%) had a mutation conferring resistance to only the non-nucleoside reverse-transcriptase inhibitors (NNRTI) class of antiretrovirals, one (14%) had mutations against nucleoside reverse transcriptase inhibitors (NRTI), while four (57%) had multiclass drug resistance against both NRTI and NNRTI drugs. Among the nine HIV-infected infants exposed to maternal NFV, all nine (100%) had an NRTI mutation, but none had a major PI mutation or NNRTI mutation (Table 2).

**Table 2:** HIV drug resistance mutations among breastfeeding infants at 6 mo post partum according to maternal regimen.

HIV Drug Resistance Mutations	Maternal Antiretroviral Therapy Regimen	
	ZDV, 3TC, NVP (*n* = 287)	ZDV, 3TC, NFV (*n* = 200)
HIV-PCR positive by 6 mo	15	9
Total with HIV drug resistance mutations	7	9
NRTI resistance	5	9
NNRTI resistance	6	0
PI resistance	0	0

One mother was originally taking NVP/ZDV/3TC, but was changed to NFV/ZDV/3TC at week 14.

Of the 12 infants who were HIV-PCR positive at delivery, 11 (92%) infants developed HIV drug resistance mutations by 6 mo. Of the 12 infants who were first HIV-PCR positive from 2 wk to 6 mo of age, five (42%) infants developed HIV drug resistance mutations by 6 mo, and none of the eight infants first positive from 6 to 24 mo developed resistance mutations. Of the 16 infants with HIV drug resistance mutations at 6 mo, 13 (81%) had at least one NRTI resistance mutation (M184 I/V [*n* = 12], K65R [*n* = 2], and D67G [*n* = 1]), and six (38%) had NNRTI resistance mutations (K103N [*n* = 2], Y181 [*n* = 2], and G190A [*n* = 2]) (sequences submitted to GenBank, http://www.ncbi.nlm.nih.gov/Genbank/index.html, accession numbers HM164112-HM164123, HM164127-HM164128, and HM164130-HM164131) (Table 3). No primary PI mutations were detected in any infant.

**Table 3:** HIV drug resistance mutation patterns among breastfeeding infants at time of emergence.

Infant Number	Mother's Regimen	Time of First Positive HIV-PCR	Time of Resistant Emergence	Mutations at Emergence	Subtypes
1-0079-3	NVP/ZDV/3TC	0–1 wk	6 wk	Y181C + G190A	A/D
1-0195-6	NVP/ZDV/3TC	0–1 wk	6 mo	K103KN	A
1-0212-2	NVP/ZDV/3TC	6 mo	6 mo	Y181C	A
1-0230-2	NVP/ZDV/3TC	0–1 wk	14 wk	M184V	D/C
1-0472-8	NVP/ZDV/3TC	0–1 wk	14 wk	K65R, M184V, K103N, Y181C	A
1-0066-8	NVP/ZDV/3TC	0–1 wk	14 wk	K65R + D67G + G190A	A
1-0317-8	NVP/ZDV/3TC	14 wk	14 wk	M184I +Y181C	D
1-0289-1	NFV/ZDV/3TC	2 wk	14 wk	M184V	A
1-0410-4	NFV/ZDV/3TC	6 wk	6 wk	M184I/V	A/D
1-0357-5	NFV/ZDV/3TC	0–1 wk	14 wk	M184V	A
1-0457-9	NFV/ZDV/3TC	0–1 wk	6 wk	K65R + M184V	A
1-360-0	NFV/ZDV/3TC	0–1 wk	6 wk	M184V	A
1-0437-5	NFV/ZDV/3TC	14 wk	14 wk	M184I	A
1-0496-6	NFV/ZDV/3TC	0–1 wk	6 wk	K65R + M184V	A
1-0517-4	NFV/ZDV/3TC	0–1 wk	14 wk	M184V	A
1-0278-8	NFV/ZDV/3TC	0–1 wk	14 wk	M184V	A/D

Phylogenetic analysis of the 32 mother–infant pairs demonstrated subtype distribution as subtype A1 (65.6%), subtype D (9.4%), subtype C (3.1%), and inter-subtype recombinants (21.9%). HIV-1 subtypes among the infants with HIV drug resistance mutations were as follows: 11 (69%) pure subtype A1, one (6%) subtype D, four inter-subtype recombinants (25%). Of the four recombinants, three were circulating recombinant forms (CRFs) A/D and one was CRF D/C. Co-relation between the mother–infant pair sequences was high with bootstrap values of >98% and nucleotide similarity of >97%.

### Assessment for the Presence of Transmitted HIV Drug Resistance Mutations

Among the 24 mothers whose infants were first HIV-PCR positive by 6 mo, ten had undetectable viral load (2.6 log copies/ml) at 6 mo post partum and 14 had detectable viral load (median, 3.2 log copies/ml; range 1.7–5.9). Among these 14 women, 10 (71%) had no HIV drug resistance mutations and four (29%) had HIV drug resistance mutations (sequences submitted to GenBank, http://www.ncbi.nlm.nih.gov/Genbank/index.html, accession numbers HM164124-HM164126 and HM164129). Two of the infants born to the four mothers with drug resistance mutations had infections with wild-type strain at first time of positivity, one had HIV drug resistance mutations that differed from that of the mother, and one had overlapping mutation patterns with that of the mother (6.25%) (Table 4). Of the 24 infants first HIV-PCR positive by 6 mo, only two were on antiretrovirals for treatment before 6 mo and both had developed drug resistance mutations before initiation of antiretroviral therapy.

**Table 4:** Comparison of infant and maternal HIV drug resistance mutation patterns by 6 mo.

Infant Number	HIV Drug Resistance Mutations			
	Maternal		Infant	
	NRTI	NNRTI	NRTI	NNRTI
0–0085	—	K103N	—	—
0–0158	—	K103N	—	—
0–0195	—	V106A	—	K103N
0–0472	M184V	K103N	M184V, K65R	K103N, Y181C

## Discussion

In this study, we investigated the emergence of drug resistance among HIV-infected breastfeeding infants and found that two-thirds of infants who became infected before 6 mo developed resistance to one or more antiretroviral drugs, most likely because of exposure to the drugs through the mother's breast milk. Previous studies have shown that taking antiretroviral therapy while breastfeeding significantly reduces the chances that HIV-infected mothers will transmit the virus to their infants [15],[17],[21]–[26]. However, the infants who get infected (and not put on antiretroviral therapy soon enough or left untreated) may be at risk of developing resistance to some of the antiretroviral drugs the mother is taking. The patterns of HIV viral load in infants and the timing of detection of HIV drug resistance mutations suggests that drug-resistant HIV variants were likely not transmitted. As previously reported by Mirochnick and colleagues [13], 3TC and NVP given to mothers are transmitted to their infants via breastfeeding in quantities sufficient to have biologic, but suboptimal, effect on HIV with potential risk of emergence of HIV drug resistance [13]. Thus, the HIV drug resistance mutations reported here likely emerged as a result of exposure to antiretroviral drugs ingested by the infant either through single-dose NVP exposure at delivery or indirectly through ingestion of antiretroviral drugs through breast milk. This possibility has also been suggested by the findings of the 6-wk extended NVP study (SWEN) in Uganda, which found the presence of NRTI resistance in infants provided with NVP prophylaxis while the mothers were on a triple-antiretroviral regimen [27].

Fourteen infants were first HIV-PCR positive by week 2. Only eight of the 14, however, had detectable viral load and none of these had HIV drug resistance mutations detected at the time of diagnosis of HIV infection. The frequency of HIV drug resistance mutations increased over time; 30% of HIV-positive infants developed drug resistance mutations by week 6, 63% by week 14, and 67% by 6 mo. With only one mother–infant pair having similar HIV drug resistance patterns and no cases of HIV drug resistance mutations among the infants first HIV-PCR positive after the mothers discontinued antiretrovirals, there is little evidence to suggest that mothers transmitted resistant HIV to their infants. With single-dose NVP, Eshleman and colleagues [22] reported a 46% rate of NVP-resistant mutations at 6 wk, and 55% by 12–14 wk, with subsequent waning by 12 mo using standard ViroSeq genotyping techniques. In the present study, where mothers received triple-antiretroviral regimen through 6 mo of breastfeeding and infants chronically ingested suboptimal quantities of antiretroviral drugs via breast milk, there were increased rates of HIV drug resistance mutations in infants at 14 and 24 wk compared to 6 wk, with no evidence of fading of HIV drug resistant mutations. It is most likely that the resistance was maintained over time in the HIV-infected infants because of ongoing exposure to low quantities of antiretroviral drugs in the mother's breast milk.

Our data illustrate a possible relationship between higher viral load in the HIV-infected infants and emergence of drug resistance mutations. The viral load at birth was likely partially suppressed owing to the maternal antiretroviral therapy transferred through the placenta [13]. The decrease in viral load from delivery to 2 wk is presumably due to the single-dose NVP given to all infants at birth and the sharp increase in viral load from 2 wk to 6 wk coincides with the emergence of HIV drug resistance mutations from 6 wk to 6 mo (Figure 2).

Kenyan National guidelines and WHO guidelines for early infant diagnosis recommend testing for HIV at 6 wk [23]. With wide-scale implementation of early infant HIV diagnosis, breastfeeding HIV-infected infants could be started on antiretrovirals different from the maternal antiretrovirals to reduce risk of development of HIV drug resistance and, therefore, lower morbidity and mortality among the HIV-infected infants [24]. It is possible that infection in the first few weeks (not detected by DNA PCR) could still be affected by drug pressure from the infant NVP dose, which could contribute to the observed NVP resistance at 6 wk.

One major limitation in this study was that HIV drug resistance genotyping was performed on maternal plasma samples and not on breast milk, which is a relatively secluded biologic compartment with differences in drug concentrations and may possibly have had drug resistance HIV variants that could be passed to the infants; however, genotypic results from the majority of the infants at the first time of diagnosis revealed the absence of resistant strains. We previously showed that drug concentrations in breast milk and bloodstream differ, and that biologically significant, but suboptimal, amounts of antiretrovirals for treatment of HIV infection could be passed to the infant via breastfeeding [13]. Breast milk concentrations of NVP were ∼70% of maternal plasma levels and in the infants were below the accepted target trough concentration for treatment of HIV. 3TC concentrations were ∼2.5-fold higher in the breast milk than maternal plasma, but below optimal plasma concentrations in the infant. The high proportion of infants developing 3TC resistance mutations in this report is consistent with the sub therapeutic concentrations of 3TC. The relatively low frequency of 3TC resistance among HIV-infected infants exposed to maternal NVP/ZDV/3TC suggests that NVP may be preventing or delaying emergence of 3TC resistance. In contrast, the fact that all HIV-infected infants exposed to maternal NFV/ZDV/3TC developed a 3TC mutation may be explained by possible low levels of NFV in breast milk and consequently low quantities transmitted to the infant [25]. The appearance of K65R in one-quarter of the infants with resistance (Table 3) is of concern, and this might be due to a high level of 3TC in the breast milk [13],[19]. A follow-up genotypic analysis on the detection of K65R mutation in breast milk is necessary. ZDV concentrations were very low in breast milk, and, in the infants, were below the limit of quantification most of the time. None of the infants had mutations associated with ZDV resistance, which appears consistent with the low infant plasma ZDV concentrations detected [13].

All infants in the KiBS trial received single-dose NVP prophylaxis at birth; however, no infants exposed to maternal NFV/ZDV/3TC developed NVP drug resistance mutations. This finding suggests that this maternal regimen provided an effective “tail” with 3TC via breast milk to reduce emergence of NVP drug resistance mutations in the infants during the first few weeks when infant NVP drug levels following single-dose NVP were still present. In contrast, six of 15 infants exposed to both single-dose NVP as well as maternal NVP through breast milk developed at least one NNRTI mutation after 2 wk of age. This finding corroborates other reports that showed that extended infant NVP prophylaxis for PMTCT significantly reduces transmission of HIV to infants [21],[26], but with a high risk of developing resistance to NVP [27],[28]. Notably, no infants exposed to maternal NFV developed drug resistance mutations which is consistent with unpublished findings in a subset of women from the KIBS study that NFV and its active metabolite (M8) transfer into breast milk in low concentrations and the resulting concentrations in dried blood spots of their breast-feeding infants for NFV were less than the limit of quantification (LOQ)−30ng/ml, and for M8 <LOQ −32ng/ml [Weidle PJ, personal communication]. In another study, NFV was present in extremely low concentrations in breast milk and M8 was not detected [29]. Others have found no detectable NFV in the plasma of breastfeeding infants whose mothers were taking NFV as part of triple-combination antiretroviral therapy [25]. The subtype distribution observed in this study follow a typical pattern of viruses circulating in this region [30]. Furthermore, we did not observe preferential selection of resistance in these infants based on subtypes.

The WHO recommends exclusive breastfeeding for HIV-exposed infants in resource-limited settings where acceptable, feasible, affordable, sustainable, and where safe replacement feedings cannot be achieved [31]. Owing to this recommendation, there is an urgent need to assess risks and benefits of antiretroviral drugs that are less likely to be transmitted through breast milk, thus reducing risk of exposure of infants to subtherapeutic levels of antiretroviral drugs and hence reducing the risk of development of HIV drug resistance among infants who become HIV infected [13]. The differential development of drug resistance mutations in the infant depending on the maternal antiretroviral regimen may have implications for subsequent treatment options. This tradeoff for low maternal-to-child transmission rates with the use of triple-combination maternal antiretroviral therapy, which includes NVP during breastfeeding, may require more complicated treatment regimens (e.g., including a PI) to treat the small number of infants who do become HIV infected in order to reduce development of drug resistance. However, the options for antiretroviral regimens available in most resource-limited settings are quite limited and so may forestall introduction of ideal combinations that address our findings. Because HIV drug resistance mutations in most infants emerge over time, improvements in early infant HIV diagnosis and treatment programs could mitigate the problem [24] before drug resistance mutations develop from exposure to maternal antiretrovirals through breastfeeding.

In conclusion, the low mother-to-child HIV transmission rates observed in the KiBS trial support the role of triple-combination maternal antiretroviral therapy as a successful PMTCT intervention among breastfeeding HIV-infected mothers [14]. However, the data from this secondary analysis suggest that ingestion of antiretroviral drugs through breast milk may have contributed to the emergence of HIV drug resistance mutations in the infants, as we observed an increasing frequency of infants with HIV drug resistance mutations over the first 6 mo of life when maternal antiretroviral therapy was given during breastfeeding. Infant drug resistance mutation patterns, depending on the maternal regimen, may have implications for subsequent HIV treatment of the small number of infants, exposed to these maternal regimens, who become HIV infected. Maternal antiretroviral regimens used for PMTCT among HIV-infected breastfeeding women should be evaluated to determine evolution of HIV drug resistance mutations among infants who become HIV infected while exposed to these regimens. PMTCT programs providing maternal antiretroviral therapy during breastfeeding and those caring for infants exposed to antiretroviral through breast milk will need to be cognizant of this issue and consider monitoring these infants more closely and tailoring their treatment accordingly.

## Supporting Information

Text S1 Protocol.
(1.24 MB PDF)

## Abbreviations

3TC: lamivudine
KiBS: Kisumu Breastfeeding Study
MTCT: mother-to-child transmission
NFV: nelfinavir
NNRTI: non-nucleoside reverse-transcriptase inhibitor
NRTI: nucleoside reverse transcriptase inhibitor
NVP: nevirapine
PI: protease inhibitor
PMTCT: prevention of mother-to-child transmission
ZDV: zidovudine

## References

[1] UNAIDS (2008). http://www.unaids.org/en/media/unaids/contentassets/dataimport/pub/globalreport/2008/jc1511_gr08_executivesummary_en.pdf.

[2] Harambat J, Fassinou P, Becquet R, Toure P, Rouet F (2008). 18-month occurrence of severe events among early diagnosed HIV-infected children before antiretroviral therapy in Abidjan, Cote d'Ivoire: a cohort study.. BMC Public Health.

[3] Dabis F, Ekpini ER (2002). HIV-1/AIDS and maternal and child health in Africa.. Lancet.

[4] Wiktor SZ, Ekpini E, Karon JM, Nkengasong J, Maurice C (1999). Short-course oral zidovudine for prevention of mother-to-child transmission of HIV-1 in Abidjan, Cote d'Ivoire: a randomised trial.. Lancet.

[5] Jackson JB, Musoke P, Fleming T, Guay LA, Bagenda D (2003). Intrapartum and neonatal single-dose nevirapine compared with zidovudine for prevention of mother-to-child transmission of HIV-1 in Kampala, Uganda: 18-month follow-up of the HIVNET 012 randomised trial.. Lancet.

[6] UNICEF (2005). http://www.unicef.org/aids/index.html.

[7] Heinig MJ (2002). Breastfeeding decisions.. Pediatrics.

[8] Heinig MJ (2001). Host defense benefits of breastfeeding for the infant. Effect of breastfeeding duration and exclusivity.. Pediatr Clin North Am.

[9] Heinig MJ (1998). The American Academy of Pediatrics recommendations on breastfeeding and the use of human milk.. J Hum Lact.

[10] Nommsen-Rivers L, Heinig MJ (1997). HIV transmission via breastfeeding: reflections on the issues.. J Hum Lact.

[11] Moodley D, Moodley J, Coovadia H, Gray G, McIntyre J (2003). A multicenter randomized controlled trial of nevirapine versus a combination of zidovudine and lamivudine to reduce intrapartum and early postpartum mother-to-child transmission of human immunodeficiency virus type 1.. J Infect Dis.

[12] Mirochnick M, Siminski S, Fenton T, Lugo M, Sullivan JL (2001). Nevirapine pharmacokinetics in pregnant women and in their infants after in utero exposure.. Pediatr Infect Dis J.

[13] Mirochnick M, Thomas T, Capparelli E, Zeh C, Holland D (2009). Antiretroviral concentrations in breast-feeding infants of mothers receiving highly active antiretroviral therapy.. Antimicrob Agents Chemother.

[14] Thomas T, Masaba R, Ndivo R, Zeh C, Borkowf C (2011). Triple-antiretroviral prophylaxis to prevent mother-to-child HIV transmission through breastfeeding—The Kisumu Breastfeeding Study, Kenya: A Clinical Trial.. PLoS Med.

[15] World Health Organization (2003). Scaling up antiretroviral therapy in resource-limited settings.. Treatment guidelines for a public health approach.

[16] Mofenson LM, McIntyre JA (2000). Advances and research directions in the prevention of mother-to-child HIV-1 transmission.. Lancet.

[17] Dorenbaum A, Cunningham CK, Gelber RD, Culnane M, Mofenson L (2002). Two-dose intrapartum/newborn nevirapine and standard antiretroviral therapy to reduce perinatal HIV transmission: a randomized trial.. JAMA.

[18] United States Food and Drug Administration (2005). http://www.fda.gov/Drugs/DrugSafety/PostmarketDrugSafetyInformationforPatientsandProviders/DrugSafetyInformationforHeathcareProfessionals/PublicHealthAdvisories/ucm051674.htm.

[19] Johnson VA, Brun-Vezinet F, Clotet B, Gunthard HF, Kuritzkes DR (2008). Update of the drug resistance mutations in HIV-1: spring 2008.. Top HIV Med.

[20] Swofford DL (2001). PAUP: Phylogenetic analysis using parsimony, version 4..

[21] Lidstrom J, Guay LA, Musoke P, Owor M, Onyango-Makumbi C (2010). Multi-class drug resistance arises frequently in hiv-infected breastfeeding infants whose mothers initiate highly active antitetroviral therapy (HAART) post-partum [Abstract].. The 17th Conference on Retroviruses and Opportunistic Infections; 16-19 February 2010; San Francisco (California, United States).

[22] Eshleman SH, Mracna M, Guay LA, Deseyve M, Cunningham S (2001). Selection and fading of resistance mutations in women and infants receiving nevirapine to prevent HIV-1 vertical transmission (HIVNET 012).. AIDS.

[23] World Health Organization (2007). http://whqlibdoc.who.int/publications/2008/9789241596596_eng.pdf.

[24] Violari A, Cotton MF, Gibb DM, Babiker AG, Steyn J (2008). Early antiretroviral therapy and mortality among HIV-infected infants.. N Engl J Med.

[25] Corbett A, Martinson F, Rezk N, Kashuba A, Jamieson D (2008). Antiretroviral drug concentrations in breastmilk and breastfeeding infants.. The 15th Conference on Retroviruses and Opportunistic Infections; 3–6 February 2008; Boston (Massachusetts, United States).

[26] Kumwenda NI, Hoover DR, Mofenson LM, Thigpen MC, Kafulafula G (2008). Extended antiretroviral prophylaxis to reduce breast-milk HIV-1 transmission.. N Engl J Med.

[27] Moorthy A, Gupta A, Sastry J, Venkatramani V, Bbosale R (2008). Timing of infection is critical for nevirapine resistance outcomes among breastfed subtype C HIV-1 infected infants exposed to extended vs single-dose NVP prophylaxis: The India SWEN Study [Abstract 44, 86 2008].. The 15th Conference on Retroviruses and Opportunistic Infections; 3–6 February 2008; Boston (Massachusetts, United States).

[28] Church JD, Omer SB, Guay LA, Huang W, Lidstrom J (2008). Analysis of nevirapine (NVP) resistance in Ugandan infants who were HIV infected despite receiving single-Dose (SD) NVP versus SD NVP plus daily NVP up to 6 weeks of age to prevent HIV vertical transmission.. J Infect Dis.

[29] Colebunders R, Hodossy B, Burger D, Daems T, Roelens K (2005). The effect of highly active antiretroviral treatment on viral load and antiretroviral drug levels in breast milk.. AIDS.

[30] Yang C, Li M, Shi YP, Winter J, van Eijk AM (2004). Genetic diversity and high proportion of intersubtype recombinants among HIV type 1-infected pregnant women in Kisumu, western Kenya.. AIDS Res Hum Retroviruses.

[31] World Health Organization (2007). http://whqlibdoc.who.int/publications/2007/9789241595964_eng.pdf.

